# The efficacy of surgery in advanced hepatocellular carcinoma: a cohort study

**DOI:** 10.1186/s12957-020-01887-8

**Published:** 2020-06-02

**Authors:** Lei Chen, Tao Sun, Shi Chen, Yanqiao Ren, Fan Yang, Chuansheng Zheng

**Affiliations:** 1 Department of Radiology, Union Hospital, Tongji Medical College, Huazhong University of Science and Technology, Wuhan, 430022 China; 2 Hubei Province Key Laboratory of Molecular Imaging, Wuhan, 430022 China; 3 Department of Interventional Radiology, Union Hospital, Tongji Medical College, Huazhong University of Science and Technology, Wuhan, 430022 China

**Keywords:** Hepatocellular carcinoma, Surgery, Liver resection, Efficacy

## Abstract

**Background:**

It is still controversial whether hepatocellular carcinoma (HCC) patients with lymph node invasion should receive surgery treatment. This study aimed to evaluate the efficacy of surgery (liver resection and local tumor destruction treatments) in HCC patients with regional lymph node metastasis.

**Methods:**

The study utilized data from the Surveillance, Epidemiology, and End Results-18 (SEER-18) cancer registry. Patients for whom the treatment type was not clear or those with distant metastasis or without regional lymph nodule invasion were excluded. For survival analysis, patients with the survival months coded as 0 and 999 were excluded. All 1434 patients were included in the analysis. Among them, 168 patients were treated surgically and the other 1266 received non-surgery therapy. Propensity score matching (PSM) model was used to reduce selection bias.

**Results:**

Before PSM, the median overall survival (mOS) and median cancer-specific survival (mCSS) of patients treated surgically were longer than that of receiving non-surgery treatment (mOS 20 months, 95% CI 15.3–24.7 vs. 7 months, 95% CI 6.4–7.6, *P* < 0.001; mCSS 21 months, 95% CI 115.5–26.5 vs. 6 months, 95% CI 5.3–6.7, *P* < 0.001). Subgroup analysis found no significant differences in mOS and mCSS between liver resection and non-liver resection surgery cohorts (*P* = 0.886 and *P* = 0.813, respectively). Similar results were obtained in the PSM analysis. The mOS and mCSS in the surgery group were longer than those in the non-surgery group (mOS 20 months vs. 7 months, *P* < 0.001; mCSS 20 months vs. 6 months, *P* < 0.001). The multivariate analysis documented that surgery was an independent predictor for OS and CSS before and after PSM.

**Conclusions:**

HCC patients with invasion of regional lymph nodules may get more survival benefit from surgery than other types of treatment.

## Introduction

Hepatocellular carcinoma (HCC) is one of the most common and lethal cancers [1, 2]. The incidence of HCC in the USA might be double by 2030 [3]. Patients with early HCC can have a high 5-year survival rate after transplantation, or liver resection, or ablation treatment [4–6]. However, typically, about 70–80% of the patients are diagnosed with HCC in the intermediate or advanced stage for there are no obvious clinical symptoms in early-stage HCC, and the radical treatments are not suitable for these people. HCC patients with intrahepatic vascular infiltration, regional lymph nodule invasion, or metastases to distant organs, and patients with cancer-related symptoms (symptomatic tumors, Eastern Cooperative Oncology Group, ECOG 1-2) are diagnosed as advanced HCC according to the guideline, and treatment with molecular target drugs is recommended [2]. The SHARP trial demonstrated that, in comparison with placebo, sorafenib prolongs the survival of patients with advanced HCC by approximately 4 months [7]. However, many patients discontinue the therapy because they cannot bear the complications of the drugs, which limited the effectiveness; as a result, only a small fraction of patients respond to pharmacologic treatment. Therefore, it is worth considering other treatments that may prolong the survival of patients with advanced HCC.

The current guideline does not recommend surgery for the treatment of advanced HCC patients [2]. However, it has been proposed that patients with regional vascular, lymph node, or organ invasion, or limited distant metastases should not be defined as advanced cancer. Conversely, they should be considered as the stage between intermediate and late stages since they could get a better survival benefit through surgery [8]. Recent data indicate that HCC patients with regional lymph node invasion or several metastasizes might receive the survival benefit from liver resection, ablation, radiotherapy, or transarterial chemoembolization (TACE) [9–13]. Although these studies presented encouraging results regarding the efficacy of surgery in patients with advanced HCC, the insufficient number of cases included in the analysis limits the strength of the conclusions reached.

The benefit of surgery in HCC patients with regional lymph nodule invasion remains unclear. Moreover, no randomized controlled trial (RCT) was conducted to address this issue. Therefore, the goal of the present study was to analyze the efficacy of surgery on HCC patients with regional lymph nodule invasion based on records available in the Surveillance, Epidemiology, and End Results-18 (SEER-18) database.

## Methods

This study utilized the information from a publicly available cancer registry, SEER-18. The database includes approximately 28% of the US population (Connecticut, San Francisco, Iowa, Detroit, New Mexico, Alaska Native Registry, Seattle, Hawaii, Utah, Atlanta, San Jose-Monterey, rural Georgia, Los Angeles, Kentucky, New Jersey, California [excluding San Francisco, San Jose-Monterey, and Los Angeles], Louisiana, and Georgia [excluding Atlanta and rural Georgia]). SEER-18 includes information on the site and extent of disease, treatment modality, patient survival, and demographic data.

The analysis included patients aged 30–84 years, diagnosed with HCC (International Classification of Diseases for Oncology, Third Edition (ICD-O-3), histology code 8170-8175, site code C22.0 (liver)) from 2004 to 2015. Patients for whom the treatment type was not clear or those with distant metastasis (including organs and lymph nodules) or without regional lymph nodule invasion were excluded. For survival analysis, patients with the survival months coded as 0 and 999 were excluded. A total of 1434 patients (all of them were AJCC 6th IIIC, TXN1M0-T4N1M0) were included in the study, 168 of them were treated surgically (including liver resection and local tumor destruction, cryoablation, percutaneous ethanol injection, radiofrequency ablation, and other), and 1266 received non-surgical treatment (Supplementary Figure 1). In the surgery group, 4 patients received radiotherapy before the surgery and 7 patients received radiotherapy after the surgery. The characteristics of patients before propensity score matching (PSM) are listed in Table 1.

**Table 1:** The baseline characteristics of patients before PSM

Characteristics	All patients, no. (%), 1434 (100)		*P* value
	Surgery, 168 (11.7)	Non-surgery, 1266 (88.3)	
Gender			0.363
Male	135 (80.4)	1053 (83.2)	
Female	33 (19.6)	213 (16.8)	
Age at diagnosis			0.225
30–44	7 (4.2)	27 (2.1)	
45–59	72 (42.8)	499 (39.4)	
60–74	71 (42.3)	563 (44.5)	
75–84	18 (10.7)	177 (14)	
Ethnicity			0.373
White	119 (70.8)	871 (68.8)	
Black	23 (13.7)	225 (17.8)	
Others	26 (15.5)	170 (13.4)	
Marital status			0.022
Married	103 (61.3)	638 (50.4)	
Single	57 (33.9)	571 (45.1)	
Others	8 (4.8)	57 (4.5)	
AJCC T stage			< 0.001
T1	58 (34.5)	289 (22.8)	
T2	51 (30.4)	270 (21.3)	
T3	56 (33.3)	627 (49.5)	
T4	2 (1.2)	65 (5.1)	
TX	1 (0.6)	15 (1.2)	
Year of diagnosis			< 0.001
2004–2006	49 (29.2)	178 (14.1)	
2007–2009	49 (29.2)	249 (19.7)	
2010–2012	28 (16.6)	393 (31)	
2013–2015	42 (25)	446 (35.2)	
Tumor size			0.01
≤ 3 cm	38 (22.6)	181 (14.3)	
3–5 cm	39 (23.2)	274 (21.6)	
> 5 cm	91 (54.2)	811 (64.1)	
Tumor number			0.428
1	142 (84.5)	1104 (87.2)	
2	19 (11.3)	135 (10.7)	
3	6 (3.6)	23 (1.8)	
> 3	1 (0.6)	4 (0.3)	
Radiotherapy			0.115
Yes	11 (6.5)	132 (10.4)	
No	157 (93.5)	1134 (89.6)	
Chemotherapy			0.046
Yes	77 (45.8)	684 (54)	
No	91 (54.2)	582 (46)	
Liver resection	69	–	–
Wedge or segmental resection	27	–	
Lobectomy	25	–	
Extended lobectomy	14	–	
Hepatectomy	3	–	
Non-liver resection	99	–	–
Local tumor destruction	30	–	
Cryoablation	2	–	
Percutaneous ethanol injection	5	–	
Radiofrequency ablation	55	–	
Others	7	–	

### Study outcomes

The endpoint of this study was patient death. The overall survival (OS) of patients was defined from the time of HCC diagnosis to death. Cancer-specific survival was defined as the time from HCC diagnosis to death caused by cancer.

### Statistical analysis

The data were extracted using the SEER*Stat software (version 8.3.6). Categorical variables were analyzed by the chi-square test and Fisher’s exact test. OS and CSS were plotted by the Kaplan-Meier method and compared by the log-rank test. Predictors for OS and CSS were analyzed by the Cox proportional risk model. In the research, the survival time was taken as dependent variables based on the model to analyze the influence of a lot of factors on survival. To reduce collinearity caused by some factors, we did not conduct univariate analysis, but conduct multivariate analysis directly. Multivariate analysis included the characteristics of gender, age, ethnicity, marital status, American Joint Committee on Cancer (AJCC) 6th T stage, year of diagnosis, tumor size, number of tumors, and the type of treatment (Table 2).

**Table 2:** Multivariate analysis of predictors for mortality and cancer-specific death before PSM

Characteristics	Multivariate analysis			
	Overall survival, HR (95% CI)	*P* value	Cancer-specific survival, HR (95% CI)	*P* value
Gender				
Male	Reference		Reference	
Female	1.104 (0.950, 1.282)	0.196	0.982 (0.826, 1.167)	0.833
Age at diagnosis				
30–44	Reference		Reference	
45–59	1.493 (1.025, 2.173)	0.037	1.725 (1.132, 2.627)	0.011
60–74	1.487 (1.023, 2.162)	0.038	1.768 (1.160, 2.692)	0.008
75–84	1.586 (1.068, 2.357)	0.022	1.842 (1.176, 2.884)	0.008
Ethnicity				
White	Reference		Reference	
Black	0.955 (0.823, 1.109)	0.548	0.914 (0.772, 1.084)	0.302
Others	0.914 (0.773, 1.082)	0.914	0.946 (0.788, 1.136)	0.551
Marital status				
Married	Reference		Reference	
Single	1.010 (0.898, 1.135)	0.869	1.040 (0.913, 1.186)	0.554
Others	0.988 (0.749, 1.303)	0.931	1.066 (0.793, 1.433)	0.674
AJCC T stage				
T1	Reference		Reference	
T2	1.165 (0.980, 1.385)	0.084	1.230 (1.09, 1.500)	0.040
T3	1.418 (1.217, 1.652)	< 0.001	1.412 (1.189, 1.677)	< 0.001
T4	1.703 (1.294, 2.240)	< 0.001	1.881 (1.398, 2.529)	< 0.001
TX	1.443 (0.868, 2.400)	0.157	1.343 (0.729, 2.474)	0.344
Year of diagnosis				
2004–2006	Reference		Reference	
2007–2009	0.948 (0.791, 1.137)	0.564	0.973 (0.794, 1.191)	0.789
2010–2012	1.197 (1.009, 1.421)	0.039	1.175 (0.970, 1.424)	0.100
2013–2015	1.109 (0.930, 1.322)	0.251	1.070 (0.878, 1.303)	0.503
Tumor size				
≤ 3 cm	Reference		Reference	
3–5 cm	1.305 (1.080, 1.577)	0.006	1.432 (1.151, 1.781)	0.001
>5 cm	1.685 (1.392, 2.039)	< 0.001	1.857 (1.487, 2.318)	< 0.001
Tumor number				
1	Reference		Reference	
2	0.872 (0.727, 1.046)	0.141	0.740 (0.461, 1.186)	0.210
3	0.577 (0.368, 0.904)	0.016	*N*	0.879
> 3	1.528 (0.631, 3.702)	0.348	*N*	*N*
Radiotherapy				
Yes	Reference		Reference	
No	1.550 (1.281, 1.876)	< 0.001	1.524 (1.230, 1.888)	< 0.001
Chemotherapy				
Yes	Reference		Reference	
No	1.664 (1.485, 1.865)	< 0.001	1.772 (1.559, 2.015)	< 0.001
Surgery				
Yes	Reference		Reference	
No	2.118 (1.756, 2.555)	< 0.001	2.115 (1.789, 2.501)	< 0.001

Propensity score matching (PSM) was used to reduce potential confounding effects and selection bias. The characteristics of gender, age, ethnicity, marital status, AJCC 6th T stage, year of diagnosis, tumor size, number of tumors, and the type of treatment were included in PSM assessment. A total of 608 patients were generated by a 1:4 ratio matching with an optimal caliper of 0.2. The characteristics of patients after PSM are listed in Table 3.

**Table 3:** The baseline characteristics of patients after PSM

Characteristics	All patients, no. (%), 608 (100)		
	Surgery, 165 (27.1)	Non-surgery, 443 (72.9)	*P* value
Gender			0.977
Male	135 (81.8)	362 (81.7)	
Female	30 (18.2)	81 (18.3)	
Age at diagnosis			0.765
30–44	7 (4.2)	12 (2.7)	
45–59	69 (41.8)	179 (40.4)	
60–74	71 (43.1)	201 (45.4)	
75–84	18 (10.9)	51 (11.5)	
Ethnicity			0.511
White	116 (70.3)	324 (73.1)	
Black	23 (13.9)	65 (14.7)	
Others	26 (15.8)	54 (12.2)	
Marital status			0.284
Married	100 (60.6)	257 (58)	
Single	57 (34.5)	174 (39.3)	
Others	8 (4.9)	12 (2.7)	
AJCC T stage			0.635
T1	55 (33.4)	145 (32.7)	
T2	51 (30.9)	121 (27.3)	
T3	56 (33.9)	159 (35.9)	
T4	2 (1.2)	14 (3.2)	
TX	1 (0.6)	4 (0.9)	
Year of diagnosis			0.228
2004–2006	46 (27.9)	107 (24.2)	
2007–2009	49 (29.7)	116 (26.2)	
2010–2012	28 (17)	109 (24.6)	
2013–2015	42 (25.4)	111 (25)	
Tumor size			0.532
≤ 3 cm	37 (22.4)	84 (19)	
3–5 cm	38 (23)	117 (26.4)	
> 5 cm	90 (54.6)	242 (54.6)	
Tumor number			0.208
1	141 (85.5)	402 (90.7)	
2	18 (10.9)	34 (7.7)	
3	5 (3)	5 (1.1)	
> 3	1 (0.6)	2 (0.5)	
Radiotherapy			0.796
Yes	11 (6.7)	27 (6.1)	
No	154 (93.3)	416 (93.9)	
Chemotherapy			0.950
Yes	77 (46.7)	208 (47)	
No	88 (53.3)	235 (53)	
Liver resection	68		–
Wedge or segmental resection	27	–	
Lobectomy	24	–	
Extended lobectomy	14	–	
Hepatectomy	3	–	
Non-liver resection	97		–
Local tumor destruction	30	–	
Cryoablation	2	–	
Percutaneous ethanol injection	5	–	
Radiofrequency ablation	53	–	
Others	7	–	

All statistical analyses were performed using GraphPad Prism 8.0 (GraphPad Software, San Diego, CA) and SPSS v24.0 (IBM, Chicago, IL, USA) software.

## Results

### Survival analysis

**Fig. 1:**
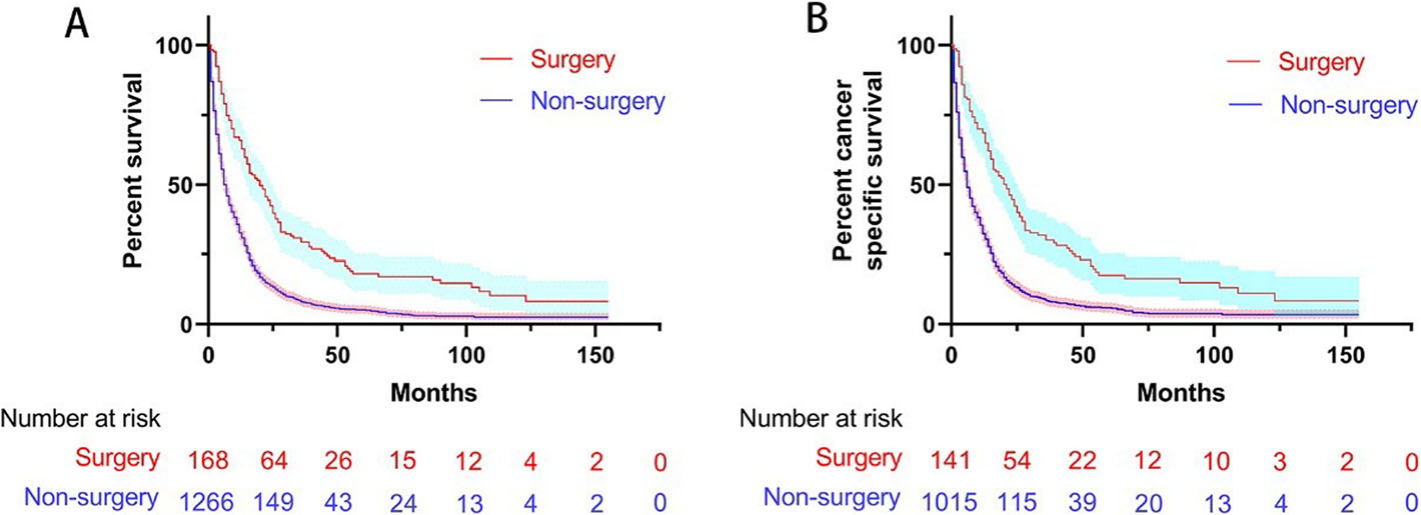
Kaplan-Meier curve of overall survival (**a**) and cancer-specific survival (**b**) of patients with surgery and non-surgery treatment before PSM; the shaded area indicates the 95% confidence interval

**Fig. 2:**
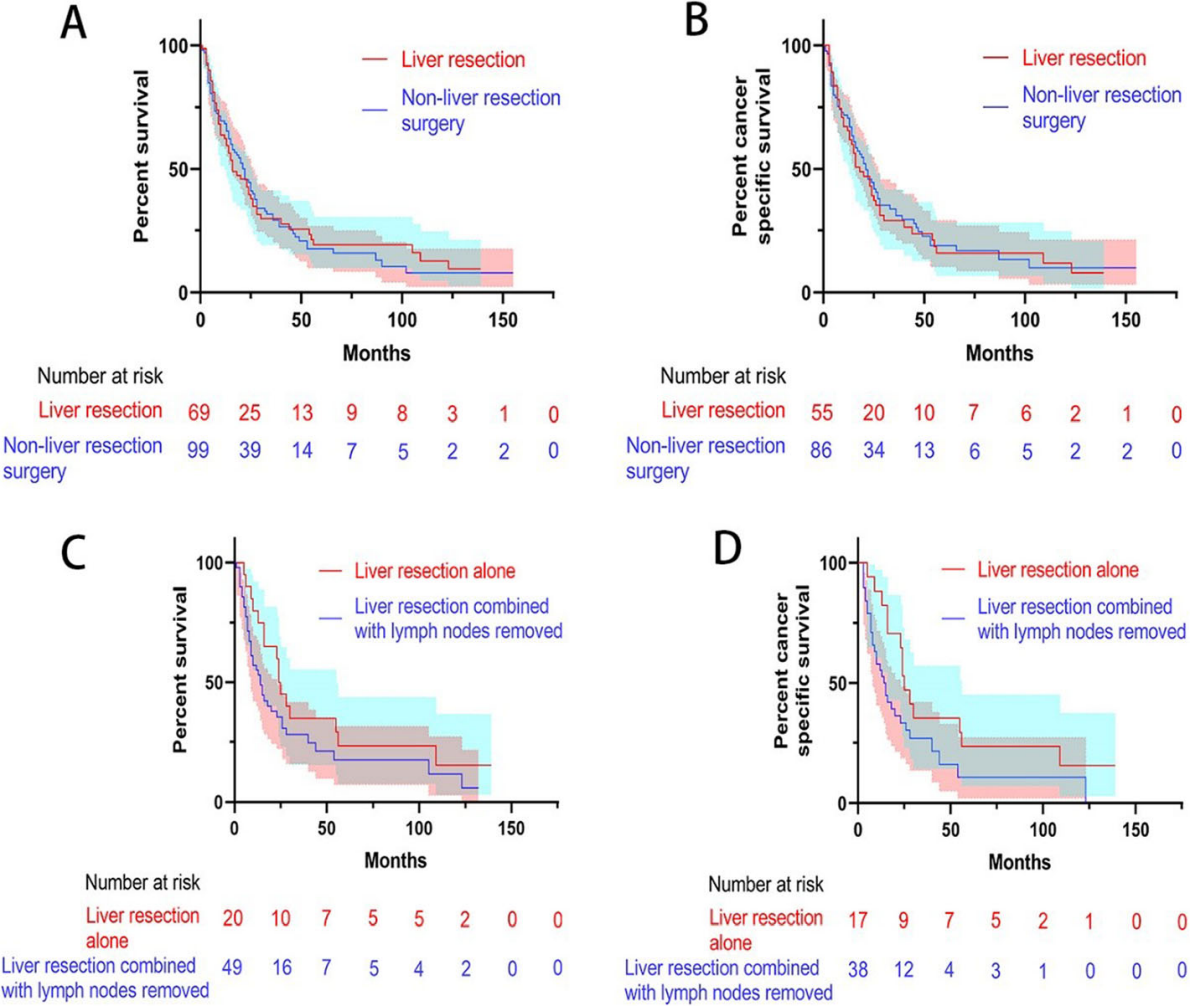
Kaplan-Meier curve of overall survival and cancer-specific survival of patients. **a** The overall survival of patients with liver resection and non-liver resection treatment. **b** The cancer-specific survival of patients with liver resection and non-liver resection treatment. **c** The overall survival of patients with liver resection alone and liver resection combined with lymph node removal treatment. **d** The cancer-specific survival of patients with liver resection alone and liver resection combined with lymph node removal treatment; the shaded area indicates the 95% confidence interval

Before PSM, the mOS in the surgery group (20 months, 95% CI 15.3–24.7) was longer than that in the non-surgery group (7 months, 95% CI 6.4–7.6), and patients with surgery had higher survival rate than patients with non-surgery (HR 0.476, 95% CI 0.397, 0.571) (Fig. 1a). In the surgery group, the subgroup analysis showed that mOS in the liver resection group (16 months, 95% CI 8.2–23.8) was similar to that in the non-liver resection group (22 months, 95% CI 16.6–27.4) (*P* = 0.886) (Fig. 2a). In the liver resection subgroup, mOS of patients with liver resection combined with the removal of regional lymph nodes (14 months, 95% CI 9.5–18.5) was similar with liver resection alone (24 months, 95% CI 21.1–26.9) (*P* = 0.142) (Fig. 2c). The mOS of patients with liver resection was longer than the mOS of patients with non-surgery (*P* < 0.001) (Supplementary Figure 2A). However, the mOS of patients with liver resection combined with the removal of regional lymph nodes was similar with the mOS of patients with non-liver resection (*P* = 0.354) (Supplementary Figure 2C).

Similar results were obtained in the analysis of CSS. The mCSS of patients treated surgically (21 months, 95% CI 15.5–26.5) was longer than in patients subjected to non-surgical treatment (6 months, 95% CI 5.3–6.7), and patients with surgery had lower cancer-specific death rate than patients with non-surgery (HR 0.475, 95% CI 0.389, 0.580) (Fig. 1b). In the subgroup analysis of the surgery group, the mCSS in patients with liver resection (18 months, 95% CI 10–26) was slightly shorter than that in patients treated without liver resection surgery (21 months, 95% CI 15.4–26.6) (*P* = 0.813) (Fig. 2b). The mCSS in patients with liver resection alone (25 months, 95% CI 20–30) was longer than with liver resection combined with lymph node removal (14 months, 95% CI 9.5–18.5) (*P* = 0.058) (Fig. 2d). The mCSS of patients with liver resection was longer than the mCSS of patients with non-surgery (*P* < 0.001) (Supplementary Figure 2B). However, the mCSS of patients with liver resection combined with the removal of regional lymph nodes was similar with the mCSS of patients with non-liver resection (*P* = 0.226) (Supplementary Figure 2D).

**Fig. 3:**
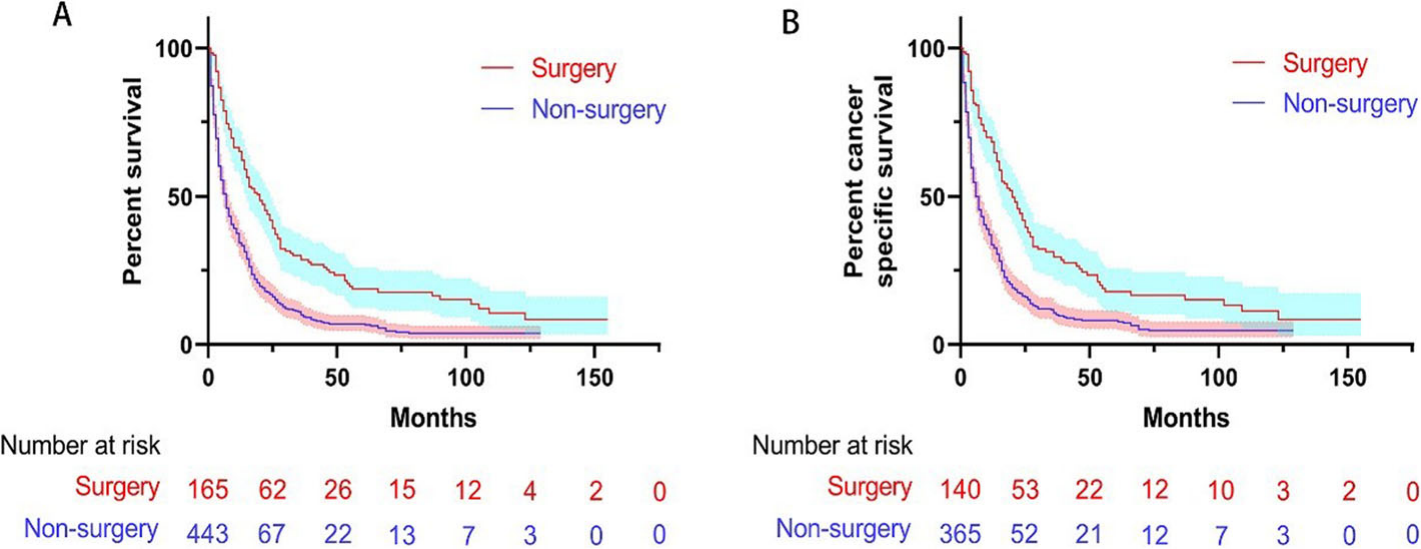
Kaplan-Meier curve of overall survival (**a**) and cancer-specific survival (**b**) of patients with surgery and non-surgery treatment after PSM; the shaded area indicates the 95% confidence interval

After PSM, the mOS and mCSS in the surgery group (mOS 20 months, 95% CI 15.1–24.9; mCSS 20 months, 95% CI 14.6–25.4) were longer than those in the non-surgery group (mOS 7 months, 95% CI 5.9–8.1; mCSS 6 months, 95% CI 4.8–7.2) (*P* < 0.001 for both mOS and mCSS) (Fig. 3a, b).

### Predictors for OS and CSS

Before PSM, the multivariate logistic regression analysis demonstrated that the treatments (non-surgery: HR 2.115; 95% CI 1.789–2.501, *P* < 0.001) were independent predictors for OS in advanced HCC patients. Older age, higher AJCC stage, larger tumor size, and without receiving radiotherapy, chemotherapy, and surgical treatment were associated with worse outcome in all patients (Table 2). For CSS, similar results were obtained in the multivariate logistic regression analysis for CSS. Older age, higher AJCC stage, larger tumor size, and without receiving radiotherapy, chemotherapy, and surgical treatment were all correlated with a shorter CSS of the patients (Table 2).

After PSM, the multivariate logistic regression analysis showed that older patients, patients with high AJCC T stage, larger tumor size, and without receiving radiotherapy, chemotherapy, and surgical treatment had worse OS and CSS (Table 4).

**Table 4:** Multivariate analysis of predictors for mortality and cancer-specific death after PSM

Characteristics	Multivariate analysis			
	Overall survival, HR (95% CI)	*P* value	Cancer-specific survival, HR (95% CI)	*P* value
Gender				
Male	Reference		Reference	
Female	1.121 (0.890, 1.411)	0.332	0.912 (0.696, 1.194)	0.501
Age at diagnosis				
30–44	Reference		Reference	
45–59	2.384 (1.369, 4.153)	0.002	2.874 (1.539, 5.365)	0.001
60–74	2.397 (1.382, 4.158)	0.002	3.055 (1.645, 5.674)	< 0.001
75–84	2.631 (1.456, 4.757)	0.001	3.059 (1.562, 5.993)	0.001
Ethnicity				
White	Reference		Reference	
Black	0.913 (0.706, 1.181)	0.490	0.844 (0.634, 1.121)	0.844
Others	0.8886 (0.675, 1.162)	0.380	0.959 (0.709, 1.296)	0.959
Marital status				
Married	Reference		Reference	
Single	0.968 (0.800, 1.172)	0.741	1.026 (0.832, 1.264)	0.812
Others	1.323 (0.792, 2.209)	0.285	1.429 (0.837, 2.442)	0.191
AJCC T stage				
T1	Reference		Reference	
T2	1.324 (1.044, 1.679)	0.021	1.381 (1.054, 1.811)	0.019
T3	1.316 (1.024, 1.691)	0.032	1.359 (1.031, 1.790)	0.030
T4	1.766 (1.028, 3.034)	0.039	1.682 (0.941, 3.008)	0.079
TX	1.462 (0.588, 3.633)	0.413	1.322 (0.478, 3.661)	0.591
Year of diagnosis				
2004–2006	Reference		Reference	
2007–2009	0.903 (0.711, 1.147)	0.219	0.852 (0.655, 1.108)	0.232
2010–2012	1.027 (0.798, 1.322)	0.834	0.932 (0.701, 1.239)	0.627
2013–2015	0.976 (0.748, 1.274)	0.858	0.886 (0.659, 1.192)	0.423
Tumor size				
≤ 3 cm	Reference		Reference	
3–5 cm	1.355 (1.040, 1.767)	0.025	1.556 (1.149, 2.107)	0.004
> 5 cm	1.940 (1.462, 2.575)	< 0.001	2.173 (1.557, 3.032)	< 0.001
Tumor number				
1	Reference		Reference	
2	0.746 (0.538, 1.035)	0.499	0.927 (0.450, 1.906)	0.927
3	0.307 (0.125, 0.754)	0.010	*N*	0.923
> 3	1.546 (0.483, 4.954)	0.463	*N*	*N*
Radiotherapy				
Yes	Reference		Reference	
No	1.765 (1.194, 2.607)	0.004	1.931 (1.262, 2.953)	0.002
Chemotherapy				
Yes	Reference		Reference	
No	1.660 (1.383, 1.991)	< 0.001	1.852 (1.510, 2.271)	< 0.001
Surgery				
Yes	Reference		Reference	
No	2.150 (1.750, 2.641)	< 0.001	2.213 (1.760, 2.781)	< 0.001

## Discussion

The most widely adopted HCC staging system is based on the Barcelona Clinical Liver Cancer (BCLC) criteria [14]. BCLC staging is endorsed by the guidelines of the American Association for the Study of Liver Diseases (AASLD) and the European Association for the Study of the Liver (EASL) due to its ability to account for liver function, tumor burden, and prognosis prediction [2, 15]. The BCLC criteria recommend that early-intermediate HCC patients with good liver function (Child-Pugh A-B) and physical condition (ECOG 0) should be treated surgically (liver resection, liver transplantation or ablation for early HCC, and TACE) [14]. However, accumulating evidence supports the conclusion that patients with intermediate HCC can obtain a better survival benefit from liver resection than from TACE [16–18], and patients with advanced HCC can get good efficacy from surgery alone or in combination with other treatments [19].

Patients with advanced HCC often have poor survival outcomes due to cancer-related impairment of liver function or physical condition. Previous studies documented that advanced HCC patients with lymph node invasion or metastases had median survival times of 6–8 months [20, 21]. However, the same group of patients treated with molecular targeted drugs, surgery alone, or a combination of both therapies could expect longer survival times of 7.0–20.4 months [7, 22–25]. In these analyses, the combination of surgery with molecular targeted drugs or with another surgery often resulted in longer mOS. Duffy and coworkers have found that patients with advanced HCC treated with the combination of tremelimumab and liver ablation had mOS of 12.3 months [26], which was longer than the mOS of patients treated with sorafenib alone [27]. A randomized phase II trial compared the efficacy of treatment by a combination of sorafenib and hepatic arterial infusion chemotherapy with that of using sorafenib alone, which has found that patients with combined therapies had longer mOS than that of single treatment [25]. The usage of surgical treatment for patients with advanced HCC is limited because it might lead to liver failure and early death. However, emergent new technologies, such as laparoscopic surgery, microwave ablation, and TACE with drug-eluting beads, limit the damage of surgery to patients and liver function. Therefore, patients with Child-Pugh A or B might get survival benefit from surgery.

The current study demonstrated that mOS of patients with surgical treatment was 20 months before PSM, which was longer than in patients treated non-surgically included in previous studies presented (mOS 7.4–7.9 months) [9, 27, 28]. Kokudo and collaborators compared the efficacy of HCC patients with portal vein tumor thrombosis who received liver resection with who received other treatments, and found that patients with liver resection had longer mOS than those not subjected to liver resection [29]. Similar results were obtained in the present study; patients with liver resection had longer mOS than patients with non-surgical treatment. However, in a subgroup analysis, mOS in patients with liver resection was comparable to that in patients undergoing procedures (such as ablation), suggesting that liver resection might not be the preferred modality in patients with regional lymph node invasion. In the liver resection group, patients with liver resection and lymph nodes resection had no longer mOS than that of liver resection alone, and there was no difference in mCSS between the two groups. Our findings suggest that in patients treated with liver resection, it should be not recommended to remove regional lymph nodes. However, the findings are hypothesis-generating rather than conclusive, and further research in this area is required. After PSM and reduction of the selection biases and confounding effects, the mOS and mCSS in the surgery group were still longer than that of the non-surgery group (*P* < 0.001), supporting the conclusion that surgical treatment for HCC patients with regional lymph node invasion could obtain a better survival benefit than non-surgical approaches.

In multivariate logistic regression analysis, age at diagnosis, year of diagnosis, AJCC T stage, tumor size, radiotherapy treatment, chemoembolization treatment, and utilization of surgery were included in the analysis to reduce mutual influence among the variables. This approach documented that non-surgical treatment was an independent unfavorable factor for OS and CSS, whether or not PSM was performed. Patients not treated with surgery would have more than 2-fold higher risk of overall death and cancer-specific death compared to patients undergoing surgery.

Liver function and physical condition of patients were not included in the current analysis as these characteristics were not recorded in the SEER database. The BCLC criteria define that patients with ECOG 1 should be classified as advanced HCC and should receive molecular targeted drugs or optimal supportive care. These patients were not included in this study, which might affect the accuracy of the results. However, patients with regional lymph node invasion were defined as having an advanced disease independently of liver function and physical condition. Thus, the study could still prove that surgery should be performed in advanced HCC patients who had good liver function and physical condition.

Although this analysis provided encouraging results, some limitations resulting from the historical design of the study should be acknowledged. First, the analysis utilized the SEER database which does not include the laboratory and imaging results; these variables might represent less precise predictors for OS and CSS. Second, selection biases may persist despite the use of the PSM model. However, PSM might be the best option to reduce selection biases since there are no RCTs or prospective studies focus on the issue presented in this analysis. Third, liver function, physical condition, whether patients received R0 resection, and that might influence the OS of patients were not incorporated in the current work, and the sample size was substantially smaller, which might lead to insufficient conclusion. Lastly, the number of invaded lymph nodules, lymphovascular invasion (LVI), and perineural invasion (PNI) which might influence the results of the study were not available and were not included in the study. Thus, we hope that future high-quality studies can confirm the findings of this study. All in all, compared with other types of treatments, patients with advanced HCC could get a better survival benefit through surgery. Lastly, despite the inclusion of many known confounders in the analysis and use of PSM, residual confounding cannot be excluded.

## Conclusions

In conclusion, although the guidelines recommend molecular targeted drugs as the first-line treatment for advanced HCC patients, among them, patients with regional lymph node invasion might benefit more from surgery than other treatments; therefore, surgery might be a better therapy option for these patients.

## Supplementary information

**Additional file 1: Figure S1**. The flowchart of patients inclusion. **Figure S2**. Kaplan-Meier curve of overall survival and cancer-specific survival of patients; A: the overall survival of patients with liver resection and non-surgery treatment; B: the cancer-specific survival of patients with liver resection and non-surgery treatment; C: the overall survival of patients with non-liver resection and liver resection combined with lymph nodes removed treatment; D: the cancer-specific survival of patients with non-liver resection and liver resection combined with lymph nodes removed treatment; the shaded area indicates the 95% Confidence Interval.

## Data Availability

The data could be found in the SEER database (https://seer.cancer.gov/data/).

## Abbreviations

HCC: Hepatocellular carcinoma
SEER: Surveillance, Epidemiology, and End Results
mOS: Median overall survival
mCSS: Median cancer-specific survival
PSM: Propensity score matching
ECOG: Eastern Cooperative Oncology Group
TACE: Transarterial chemoembolization
AJCC: American Joint Committee on Cancer
