# Supplementary material for: The efficacy of surgery in advanced hepatocellular carcinoma: a cohort study
Source: World J Surg Oncol. 2020 Jun 2;18:119. doi: 10.1186/s12957-020-01887-8 (PMC7268283; doi:10.1186/s12957-020-01887-8)
Supplement: Supplementary file 1 — Additional file 1: Figure S1. The flowchart of patients inclusion. Figure S2. Kaplan-Meier curve of overall survival and cancer-specific survival of patients; A: the overall survival of patients with liver resection and non-surgery treatment; B: the cancer-specific survival of patients with liver resection and non-surgery treatment; C: the overall survival of patients with non-liver resection and liver resection combined with lymph nodes removed treatment; D: the cancer-specific survival of patients with non-liver resection and liver resection combined with lymph nodes removed treatment; the shaded area indicates the 95% Confidence Interval. [file 12957_2020_1887_MOESM1_ESM.docx]

**Figure 1:** The flowchart of patients inclusion

**Figure 2:** Kaplan-Meier curve of overall survival and cancer-specific survival of patients; A: the overall survival of patients with liver resection and non-surgery treatment; B: the cancer-specific survival of patients with liver resection and non-surgery treatment; C: the overall survival of patients with non-liver resection and liver resection combined with lymph nodes removed treatment; D: the cancer-specific survival of patients with non-liver resection and liver resection combined with lymph nodes removed treatment; the shaded area indicates the 95% Confidence Interval

Response to Reviewers Comments

Dear Editors and Reviewers,

On behalf of all the contributing authors, I would like to express our sincere appreciations again of your letter and reviewers’ constructive comments concerning our manuscript entitled “The efficacy of Surgery in Advanced Hepatocellular Carcinoma: A Cohort Study”. Point-by-point responses to the nice editor and nice reviewers are listed below this letter.

Reviewer 1:

1. Authors state “We have tried to convert the AJCC 6th to AJCC 8th of HCC patients. However, the data provided by SSER database could not support the conversion. Then, we did not convert the AJCC 6th to AJCC 8th. We will use the lasted data to do further research if the SEER database provides the lasted AJCC staging in future study.” This is not acceptable authors will have to manually change the AJCC to 8th or ask someone to write a Excel program to convert, SEER will never do it and it is not acceptable to have different AJCC being used in the same study as this will cause confusion and readers are not expected to read all AJCC classifications to interpret results. This is must, without this study should not be accepted for publication

Response: Thank you for your comments. We mentioned “We have tried to convert the AJCC 6th to AJCC 8th of HCC patients. However, the data provided by SSER database could not support the conversion. Then, we did not convert the AJCC 6th to AJCC 8th. We will use the lasted data to do further research if the SEER database provides the lasted AJCC staging in future study” because there was missing data for us to convert AJCC 6^th^ to AJCC 8^th^ in the SEER database. We have carefully studied the AJCC 6^th^ staging and AJCC 8^th^ staging and found that the main difference of the two staging were the AJCC T1, T3 and T4. The AJCC 8^th^ T1 was divided as T1a and T1b, but the overall AJCC 8^th^ T1 was same with AJCC 6^th^ T1. The patients with multiple tumors and the largest tumor be larger than 5 cm, or patients with tumors which invaded portal vein or major vessels were defined as AJCC 6^th^ T3. However, in the AJCC 8^th^ staging, the patients with portal vein or major vessels were defined as T4. In the AJCC 6^th^ staging, the information that how many patients with the largest tumor larger than 5 cm and invaded the portal vein or major vessel was unknown, which lead that we could not convert AJCC 6^th^ to AJCC 8^th^. We do agree that there should not different AJCC staging exist in one study. As we referred that “In the SEER database, the AJCC 6^th^ and 7^th^ of hepatocellular carcinoma were provided. However, the SEER database only provided the AJCC 6^th^ of HCC from 1998 to 2015 and the AJCC 7^th^ of HCC from 2010 to 2015. In the study, we used AJCC 6^th^ as the HCC staging because the study included patients diagnosed as HCC from 2004 to 2015”, we used AJCC 6^th^ staging in the study.

2. In response to point 5, Authors state “However, in the SEER data, we could not get the information whether patients received chemotherapy before or after surgery. We will focus on the issues in the future study.” Authors should note that they will have to get all information now, if data is available they should get it, there is nothing called future study from same database on same topic.

Response: Thank you for your comments. The future study does not mean that we will conduct research based on the same database, but we will conduct the future study based on the data from our hospital or multiple hospitals.

3. Authors are unable to get information on site of node, number etc. as in 6th AJCC this was not recorded in SEER database, this is a limitation of the study and should be included in limitations.

Response: Thank you for your comments. We added the limitation in the revised manuscript.

4. Authors are unable to provide information on R0 resection, LVI and PNI due to limitation of data base, as of today we know these are important prognostic factors and there is no point of discussing anything on prognosis without so much of missing data.

Response: Thank you for your comments. We feel very sorry that some factors which might influence the results of the study could not be provided in the study because the SEER database did not provide. The information of R0, LVI and PNI was not available, which was the limitation of the study. Thus, we added the these in the section of limitation.

5. In response to point 9 authors are not able to provide all factors into prognostic evaluation, I wonder what is point of publishing analysis of data with so much of missing. Can authors justify why this article with so much of important information missing be published?

Response: Thank you for your comments. In this study, we have provided most factors we could get which might influence the results from the SEER database. One factor that patients insure was not included because that the SEER database warned that the information of patients insure might not very accurate. However, we need to explain that the superiority of SEER database is that it includes approximately 28% of United States population (Connecticut, San Francisco, Iowa, Detroit, New Mexico, Alaska Native Registry, Seattle, Hawaii, Utah, Atlanta, San Jose-Monterey, rural Georgia, Los Angeles, Kentucky, New Jersey, California [excluding San Francisco, San Jose-Monterey, and Los Angeles], Louisiana and Georgia [excluding Atlanta and rural Georgia]), which can provide massive data that are hard get from one hospital or multiple hospitals. Researches can use the data to do many researches that cannot be conducted by using the data from hospitals because the hospitals might not provide enough data to do it. And the massive data might strength the credibility of the results. The major disadvantage of the SEER database is that it cannot provide some factors which might influence the results of the study. However, the results of the study based on SEER database can provide ideals for future studies (presented in the link below). And the results of many studies based on SEER database have been proved to be correct. So, the results of this study might provide new ideals for future study and need RCT to prove.

1. Yan M, Ha J, Aguilar M, et al. Birth cohort-specific disparities in hepatocellular carcinoma stage at diagnosis, treatment, and long-term survival. J Hepatol. 2016;64: 326-332.
2. El-Serag HB, Mason AC, Key C. Trends in survival of patients with hepatocellular carcinoma between 1977 and 1996 in the United States. Hepatology. 2001;33: 62-65.
3. Yang D, Hanna DL, Usher J, et al. Impact of sex on the survival of patients with hepatocellular carcinoma: a Surveillance, Epidemiology, and End Results analysis. Cancer. 2014;120: 3707-3716.
4. Jiang YQ, Wang ZX, Deng YN, Yang Y, Wang GY, Chen GH. Efficacy of Hepatic Resection vs. Radiofrequency Ablation for Patients With Very-Early-Stage or Early-Stage Hepatocellular Carcinoma: A Population-Based Study With Stratification by Age and Tumor Size. Front Oncol. 2019;9: 113.
5. Wang X, Chen M, Wei R, Wang Z. External Radiation versus Internal Radiation for Patients with Advanced Unresectable HCC -A SEER Based Study. J Cancer. 2019;10: 1171-1180.

6. In response to point 10 authors is unable to provide what was done in non surgical group, absence of important information in control group is worrisome and will ultimately lead to biased interpretation of results.

Response: Thank you for your comments. The non-surgical group, 132 patients received radiotherapy and 684 patients received chemotherapy, which was presented in Table 1.

Once again, thank you very much for your comments and suggestions.

Best wish to you and the world
Chuansheng Zheng
Department of Radiology
Union Hospital, Tongji Medical College, Huazhong University of Science and Technology

Wuhan 430022

China
